# Prognostic Value of microRNA Signature in Patients with Gastric Cancers

**DOI:** 10.1038/srep42806

**Published:** 2017-02-16

**Authors:** Hai-Ting Liu, Ya-Wen Wang, Ai-Yan Xing, Duan-Bo Shi, Hui- Zhang, Xiang-Yu Guo, Jing- Xu, Peng Gao

**Affiliations:** 1 Department of Pathology, Qilu Hospital, Shandong University, Jinan, P.R. China; 2 Department of Pathology, School of Medicine, Shandong University, Jinan, P.R. China; 3 Department of Pathology, Qingdao Central Hospital, Qingdao, P.R. China

## Abstract

The occurrence of lymph node metastases (LNM) after endoscopic submucosal dissection (ESD) in patients with gastric cancer (GC) leads to poor prognosis. However, few biomarkers are available to predict LNM in GC patients. Thus, we measured expression of 6 cancer-related miRNAs using real-time RT-PCR in 102 GC samples that were randomized into a training set and a testing set (each, 51 cases). Using logistic regression, we identified 4-miRNA (miR-27b, miR-128, miR-100 and miR-214) signatures for predicting LNM in GC patients. Patients with high-risk scores for the 4-miRNA signature tended to have higher LNM than those with low-risk scores. Meanwhile, the ROC curve of the 4-miRNA signature was better for predicting LNM in GC patients. In addition, Cox regression analysis indicated that a 2-miRNA signature (miR-27b and miR-214) or a miR-214/N stage signature was predictive of survival for GC patients. This work describes a previously unrecognized 4-miRNA signature involved in LNM and a 2-miRNA signature or miR-214/N stage signature related to GC patients’ survival.

Gastric cancer (GC) is the second most common cause of global cancer mortality, with approximately 1 million people dying per year^1^. Early gastric cancer (EGC) is defined as a tumor of the stomach that invades the mucosa and submucosa (T1 cancer), irrespective of lymph node metastases^2^. Endoscopic submucosal dissection (ESD), as a minimally invasive treatment, is used to treat EGC^3^. However, lymph node metastases (LNM) frequently were observed after ESD in patients with EGC^4^,^5^,^6^,^7^. It remains unclear whether LNM co-occur with ESD—LNM contributes to poor prognosis with GC; so lymph node status has been identified as the most crucial prognostic factor^8^. Thus, identification of biomarkers to predict LNM for GC patients is urgently needed.

MicroRNAs (miRNAs), small non-coding RNAs consisting of approximately 22 nucleotides, degrade mRNA or down-regulate translation of target proteins^9^. Studies suggest that miRNAs contribute to tumor metastasis^10^ and are recognized to be needed for the development and progression of many cancers^11^. Research suggests that miRNA expression may be a novel diagnostic and prognostic biomarker for various cancers^12^ and miRNA signatures have been indicated to be more precise for cancer classification than mRNA profiles^13^. Several studies of miRNA expression signatures in GC suggest a correlation between miRNA expression and GC development, progression, and personalized therapy^14^,^15^. The clinical significance of miRNA signature in GC patients with accompanying LNM, however, has not been established.

Recently, several cancer-related miRNAs have been shown to be dysregulated in various cancers including miR-27b, miR-101, miR-128, miR-100, miR-145 and miR-214, and contribute to the development and progression of human cancers. To date, numerous genes have been identified to be target genes of these six miRNAs, which play important roles in multiple biological signaling pathways including cellular proliferation, invasion, metastasis, apoptosis and angiogenesis in various tumors. For example, miR-27b can suppress growth, cell migration and invasion in tumor cells by targeting multiple tumor-related genes such as Sp1^16^, EGFR and c-Met^17^. Some oncogenes associated with tumor cell proliferation, invasion and metastasis, such as Pim-1^18^ and MCL-1^19^ have been identified as targets of miR-101 in many cancers. Likewise, there is evidence that miR-128 has also an important role in regulating cell growth, invasion, metastasis and angiogenesis by targeting genes including ITGA5^20^ and VEGF-C^21^. Similarly, miR-100 can suppress invasion and metastasis in cancers by targeting multiple metastasis-related genes such as Rac1^22^ and ZBTB7A^23^. MiR-145 was demonstrated to induce apoptosis, suppress proliferation, invasion, metastasis and angiogenesis by targeting multiple cancer-related genes including IRS-1^24^, MUC1^25^, Catenin-δ1^26^ and N-cadherin^9^. As well, miR-214 inhibited tumor invasion by reducing expression of β-catenin^27^ and FGFR1^28^ in hepatocellular carcinoma cells. So, these six miRNAs play effective tumor-regulatory function by modulating multiple target genes, and newly discovered target genes are increasing. Thus, in our study, we chose these six miRNAs for investigation and analyzed any correlation between them and patient prognosis or lymphatic metastasis.

Specifically, we measured expression of the 6 miRNAs in 102 cases of GC and identified a 4-miRNA signature to predict LNM and a 2-miRNA signature or miR-214/N stage for predicting overall survival for GC patients.

## Methods

### Patients and clinical clinicopathological parameters

One hundred two formalin-fixed paraffin-embedded (FFPE) tissue samples of GC obtained from Qilu Hospital, Shandong University, Jinan, China, between 2004 and 2007, were collected for miRNA expression analysis. GC tissues were confirmed by 2 pathologists. Of the 102 patients, the median follow-up time was 63.4 months (ranged 1 to 67 months). Among the 102 participants, there were 65 patients with LNM and 37 patients without LNM. Subject characteristics are summarized in Supplementary Table 3. Methods were performed according to the approved guidelines.

### RNA extraction

Paraffin tissues were cut into 4-mm thick sections, then dewaxed, rehydrated and lightly stained with hematoxylin. Tumor tissues were inspected and microdissected with a 25 G needle under a dissecting microscope. MiRNA extraction was performed with a miRNeasy FFPE kit (Bioteke, Beijing, China) according to the manufacturer’s protocol, which isolated miRNA from FFPE tissue sections.

### Real-time quantitative reverse transcription-PCR

The reverse transcription (RT) was conducted with 100 ng total miRNA and quantitative PCR reactions were done by the 7900HT system (Applied Biosystems, Foster City, CA) using an All-In-OneTM qRT-PCR detection kit (Genecopeia, Rockville, MD) following the manufacturer’s protocol. Primers for measuring 6 miRNAs were synthesized by Genecopeia. We calculated the relative amounts of selected miRNAs using the equation 2^−ΔΔCt^. U6 and RNU44 were detected by qRT-PCR as an endogenous control. Based on the median score of individual miRNA of the GC patients, expressions of the 6 miRNAs (miR-27b, miR-101, miR-128, miR-100, miR-145 and miR-214) were classified as a high- or low-expression.

### Statistical analysis

Associations between miRNA expression and clinical characteristics were assessed with either the Student’s t test, the Chi-squared test or Fisher’s exact test. A stepwise logistic regression model was used to select predictive miRNA markers. The predicted probability of positive LNM with GC was used as a surrogate marker to establish the ROC curve. The area under the curve (AUC) was used as an accuracy index for evaluating the predictive performance of the selected miRNA signature. Univariate Cox regression analysis was used to evaluate the hazard ratio (HR) of miRNA and clinical variables for patient survival. Multivariate Cox regression analysis was conducted to test for independent prognostic factors of OS. A prognostic score model was constructed to compare the miRNA signature prognostic validity with the individual miRNA model using ROC analysis. Overall survival (OS) was defined as the length from the operation date to the date of death or the final follow-up. The Student’s t test, the Chi-squared test or Fisher’s exact test were performed using the statistical software Prism 5 (GraphPad Software, La Jolla, CA), and all the other statistical tests were performed with SPSS version 20.0. Statistical significance was defined *p* < 0.05.

## Results

### Cancer-related miRNA expression of the GC training set

**Figure 1:**
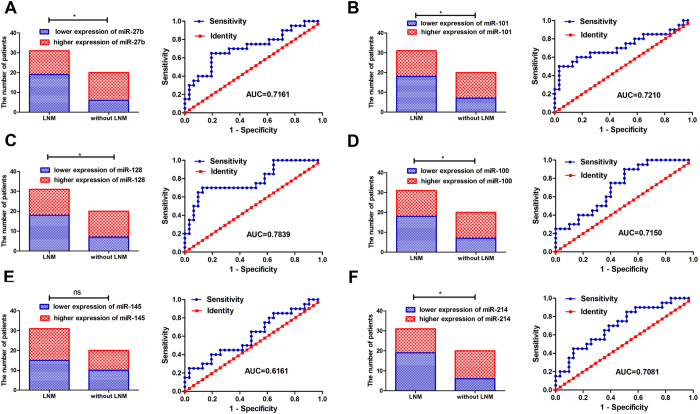
Expression of six cancer-related miRNAs and accuracy for predicting LNM in GC training sets. Expression of miR-27b (**A**), miR-101 (**B**), miR-128 (**C**), miR-100 (**D**) and miR-214 (**F**) in patients with LNM were significantly lower than for patients without LNM (all *p < *0.05). No correlation was found between expression of miR-145 (**E**) and LNM (*p > *0.05). AUC for miRNAs. (**A**) miR-27b (p = 0.0097); (**B**) miR-101 (p = 0.00824); (**C**) miR-128 (p = 0.00069); (**D**) miR-100 (p = 0.0106); (**E**) miR-145 (p = 0.1649); (**F**) miR-214 (p = 0.0128).

Specimens (N = 102) randomized into a training and a testing set (both, N = 51). In the training set, of the 6 miRNAs, miR-27b, miR-101, miR-128, miR-100 and miR-214 in expression for patients with LNM was significantly lower than for patients without LNM (Fig. 1), indicating a correlation between expression of 5 miRNAs and LNM. No correlation was found between miR-145 expression and LNM. Additionally, lower expression of miR-101, miR-128, miR-145 and miR-214 was associated with larger tumor size (p = 0.0395, p = 0.0179, p = 0.00395, and p = 0.0008, respectively). According to the American Joint Committee on Cancer^29^, tumor, node and metastasis (TNM) classification T stage was categorized into T1a (mucosa), T1b (submucosa), T2 (muscularis propria), T3 (subserosa) and T4 (tumor invades adjacent structures). A correlation was found between less miR-145 or miR-214 and positive T stage of the TNM classification (p = 0.0365 and p = 0.0203, respectively). To discover LNM-specific miRNAs in GC, the predictive accuracy of one single miRNA to distinguish between patients with and without LNM was assessed using a receiver operating characteristic (ROC) curve. Considered individually, apart from miR-145 (Fig. 1E; AUC = 0.6161, p = 0.1649), miR-27b, miR-101, miR-128, miR-100, and miR-214 had high AUC (Fig. 1A–D,F). A correlation between expression of 6 miRNAs and patient clinicopathologic characteristics appear in Supplementary Table 1.

### Validation of cancer-related miRNAs in the testing set and for both GC sets

**Figure 2:**
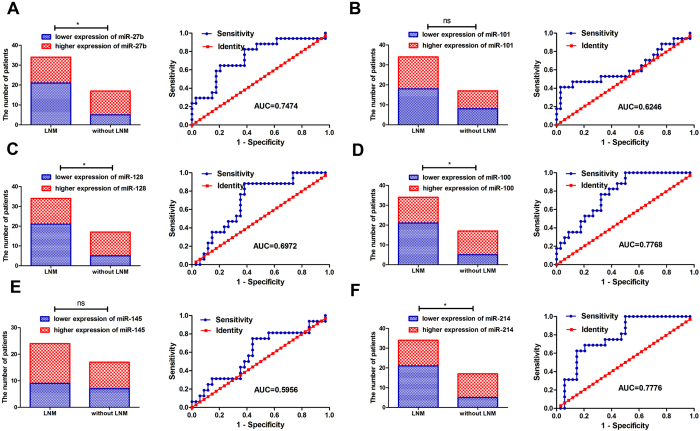
Expression of six cancer-related miRNAs and accuracy for predicting patients with LNM in GC testing sets. Expression of miR-27b (**A**), miR-128 (**C**), miR-100 (**D**) and miR-214 (**F**) in patients with LNM were significantly lower than for patients without LNM (all *p < *0.05). No correlation was found between expression of miR-101 (**B**) and miR-145 (**E**) and LNM (all *p > *0.05). AUC for the miRNAs. (**A**) miR-27b (p = 0.0043);(**B**) miR-101 (p = 0.1503); (**C**) miR-128 (p = 0.0228); (**D**) miR-100 (p = 0.0014); (**E**) miR-145 (p = 0.2795); (**F**) miR-214 (p = 0.0017).

**Figure 3:**
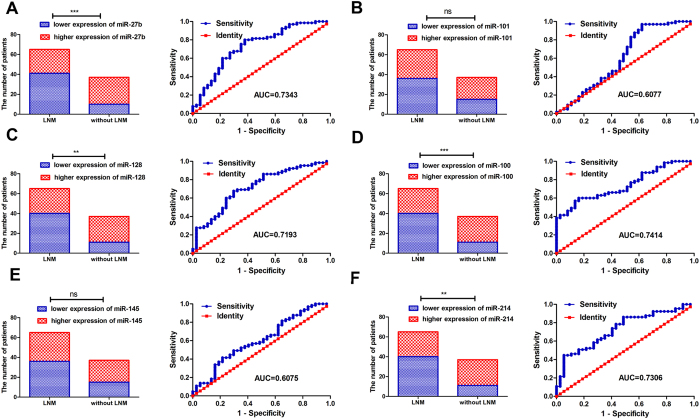
Expression of six cancer-related miRNAs and accuracy for predicting patients with LNM from both GC sets. Expression of miR-27b (**A**), miR-128 (**C**), miR-100 (**D**) and miR-214 (**F**) in patients with LNM were significantly lower than for patients without LNM (all *p < *0.05). No correlation was found between expression of miR-101 (**B**) and miR-145 (**E**) and LNM (all *p > *0.05). AUC for the miRNAs. (**A**) miR-27b (*p < *0.0001); (**B**) miR-101 (p = 0.0026); (**C**) miR-128 (p = 0.0002); (**D**) miR-100 (*p < *0.0001); (**E**) miR-145 (p = 0.0720); (**F**) miR-214 (p = 0.0001).

**Figure 4:**
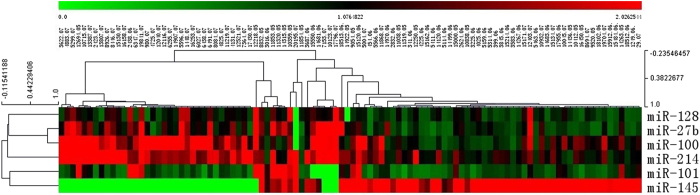
Expression changes in individual miRNA in GC samples with and without LNM. Heat map diagram: red represents overexpression; green represents underexpression; black represents unchanged.

We measured expression of 6 miRNAs in the testing set and in both GC sets and miR-27b, miR-128, miR-100 and miR-214 for the patients with LNM were significantly lower than for patients without LNM (Figs 2 and 3). No association was found between expression miR-101 or miR-145 and LNM. Additionally, to validate potential miRNAs to predict LNM in GC patients, the ROC curve in the testing set was investigated and results show that miR-27b, miR-128, miR-100, and miR-214 (Fig. 2A,C,D and F) had significantly high AUC scores excluding miR-101 and miR-145 (Fig. 2B and E). Distribution of the expression of 6-miRNAs in the 65 GC samples with LNM and 37 GC samples without LNM (training and testing sets) by hierarchical clustering showed a relative separation between the two groups apart from miR-101 (Fig. 4). Expressions of miR-27b, miR-128, miR-100 and miR-214 were down-regulated in GC samples with LNM; but in GC samples without LNM, expression of the 4 miRNAs increased. Although clustering of miR-145 indicated a relative separation, its expression was not correlated with LNM (Figs 1E,2E and 3E). Therefore, miR-101 and miR-145 were eliminated from the candidate miRNAs. In conclusion, ROC curve analysis in the training and testing sets and clustering analysis indicated that miR-27b, miR-128, miR-100, and miR-214 had stable predictive value for LNM. Associations between the 6 miRNA expression and patient clinicopathologic characteristics in the testing set are shown in Supplementary Table 2.

In addition, the association between the 4 miRNA and GC patients with LNM in combination of the two sets was demonstrated by ROC curve. Our data revealed that miR-27b, miR-128, miR-100 and miR-214 had dramatically high AUC scores (Fig. 3A,C,D,F). Thus, a combination (miR-27b, miR-128, miR-100 and miR-214) was selected to achieve the best prediction of LNM development of GC patients. The correlation between 6 single miRNA expression and patient clinicopathologic characteristics using the 2 sets is shown in Supplementary Table 3.

It was reported that RNU44 was more suitable than U6 as the endogenous control to normalize relative expression of miRNAs^30^,^31^. So, we analyzed the relationship between 6 miRNAs and LNM based on expression of 6 miRNAs normalized by RNU44. Expression of miR-27b, miR-128, miR-100 and miR-214 in patients with LNM were significantly lower than for those without LNM. No correlation was found between expressions of miR-101 or miR-145 and LNM. In addition, the ROC curve demonstrated that 4 miRNAs (miR-27b, miR-128, miR-100, and miR-214; Supplementary Figure 1A,C,D and F) had significantly high AUC scores except miR-101 and miR-145 (Supplementary Figure 1B and E). These data agree with results using U6 as a normalizer.

### A 4-miRNA signature for LNM prediction was constructed by logistic regression modeling

To assess the value of the 4 miRNAs as a predictor for LNM in GC patients, we integrated them and constructed a predictive classifier for the 102 GC patients. Logistic regression modeling was used to analyze associations with LNM. A formula was derived to calculate the miRNA score for each patient based on individual expression of the 4 miRNAs, weighted by regression coefficient, as follows:

miRNA score = (12.92 × miR − 27b expression) + (3.686 × miR − 100 expression) − (0.972 × miR − 128 expression) + (1.789 × miR − 214 expression) − 1.5

**Figure 5:**
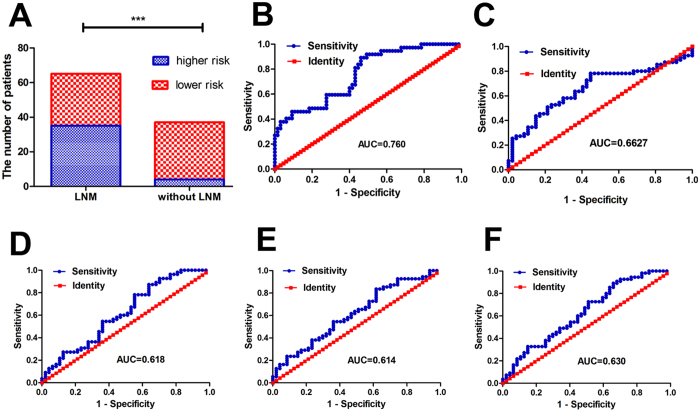
(**A**) Patients with high risk-scores tended to have higher LNM in GC patients than did those with low risk scores. (**B**) The four-miRNA signature can predict LNM in GC patients with higher AUC (*p* < 0.0001), indicating the considerable clinical value to predict LNM in GC patients. (**C**) miR-214/N stage signature can predict survival in GC patients with higher AUC (p = 0.00477), indicting the miR-214/N stage signature had better prognostic value than that of miR-214 or N stage alone for patients with gastric cancer. The combination of miRNAs (miR-27b and miR-214) (**F**, p = 0.0243) better predicted survival than miR-27b (**D**, p = 0.0403) and miR-214 (**E**, p = 0.0480) with respect to OS.

To validate the 4-miRNA signature for predicting LNM for patients with GC, patients were classified into high- or low-risk groups based on a threshold of −1.237, the maximum value of sensitivity and specificity plus 1. As expected, patients with high-risk scores tended to have higher LNM than did those with low-risk scores (Fig. 5A, *p < *0.0001). We assessed the predictive accuracy of the 4-miRNA-based classifier with ROC analysis and the miRNA marker set can predict LNM in GC patients as indicated in Fig. 5B and Supplementary Table 4, which was superior to that of the individual miRNA markers. Additionally, compared with patients in the low-risk group, patients in the high-risk group had advanced T and N stages (Table 1). Subsequently, training or testing set subjects were classified into high- or low-risk groups. In the training set, compared with the low-risk group, the high-risk group had higher LNM and larger tumor size (Supplementary Table 6). Additionally, in the testing set, patients in the high-risk group had increased LNM and advanced N stage compared with the low-risk group (Supplementary Table 7).

### A 2-miRNA signature and miR-214/N stage for survival prediction was established by Cox regression model

**Figure 6:**
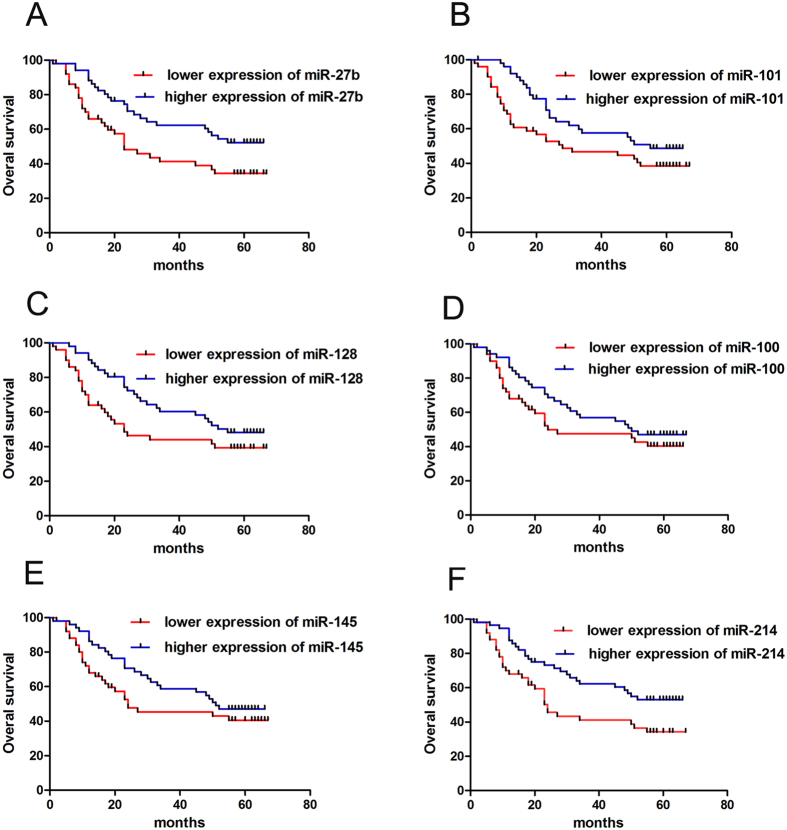
Expression of six cancer-related miRNAs and correlation with GC patient survival. Kaplan-Meier survival analysis and log-rank test showed that patients with lower miR-27b (**A**) and miR-214 (**F**) had a poorer OS. However, there was no significant difference in OS between the miR-101 (**B**), miR-128 (**C**), miR-100 (**D**) and miR-145 (**E**) lower and higher expression groups.

To investigate whether individual miRNA markers can predict GC patient survival, we used combined samples of training and testing cohorts and data show that less expression of miR-27b and miR-214 were correlated with poorer overall survival (OS) (Fig. 6A and F). However, there was no significant association between expression of miR-101, miR-128, miR-100 or miR-145 and OS (Fig. 6B–E; *p* > 0.05). To investigate whether single miRNA markers may function as an independent prognostic factor for OS in GC, we evaluated the association between the 6 miRNAs or clinicopathological parameters and prognosis by Cox regression analysis. Univariate Cox regression confirmed that miR-27b, miR-214 and 4 clinical features, such as LNM, tumor size, clinical stage and TNM, were significant predictors (Table 2). Moreover, multivariate Cox regression confirmed that miR-214 and N stage (TNM classification) were independent prognostic factors for OS (Table 2). Next, to identify a comprehensive predictive value of miR-27b and miR-214, we integrated the 2 miRNAs into a comprehensive factor using Cox regression analysis to develop a new risk score formula:

Risk score = 0.655 × expression of miR-27b + 0.687 × expression of miR-214, weighted by regression coefficient.

Using ROC analysis to compare the accuracy of survival prediction, the established miRNA panel better predicted survival than did the individual markers with regard to OS (Fig. 5D–F, Supplementary Table 5). In conclusion, the 2-miRNA signature had better prognostic value than that of individual miRNA markers for GC patients.

Next, we analyzed a combination of low expression of miR-214 and N stage with patient survival. We integrated the miR-214 and N stage into a comprehensive factor by Cox regression analysis to develop a new risk score formula: Risk score = 0.566 × the expression of miR-214 + 0.711 × N stage (N0 = 0; N1, N2 or N3 = 1), weighted by regression coefficient. The established miR-214/N stage signature better predicted survival than did the individual marker with regard to OS (Fig. 5C, Supplementary Table 8). Thus, the miR-214/N stage signature was better for prediction than the miR-214 or N stage alone for GC patients.

## Discussion

LNM is a major determinant of disease recurrence after patients undergo ESD for early gastric carcinoma^8^. However, whether the patients with early gastric carcinoma after ESD were concomitant with LNM remains unknown. For the present, there were few molecular biomarkers to predict LNM in patients with gastric carcinoma. An overwhelming majority of studies showed that mRNA expression signatures may function as prognostic biomarkers in various cancers. Thakkar *et al*. showed that over-expression of 3-gene signature (GATA3/NTN4/MLPH) enhanced prediction of relapse-free survival in ERα (+) and node (+) breast cancers^32^. Wang *et al*. also indicated that high expression of the 5-gene signature was strongly correlated with patients’ poor prognosis, and may be potential prognostic predictors in GC^33^. However, miRNA profiles have been indicated to have greater utility than mRNA profiles as prognostic biomarkers on account of miRNA stability within clinical specimens^34^. Differential miRNA expression signatures have been explored in various human cancers and miRNA expression alterations were associated closely with progression and prognosis of human malignant cancers^35^,^36^. We hypothesized that miRNA signatures may predict LNM and GC patients’ prognosis.

In this study, we describe associations between aberrant expression of specific miRNA signatures and LNM and miRNA signatures and OS of GC patients. We developed a 4-miRNA signature (miR-27b, miR-128, miR-100 and miR-214) related to LNM. Patients with high-risk scores of this 4-miRNA signature tended to have higher LNM than did those with low-risk scores. We assessed the predictive accuracy of the 4-miRNA based classifier with ROC analysis. The ROC curve of the 4-miRNA signature had higher AUC than did individual miRNA markers. In addition, our results showed that the 4-miRNA-based classifier can predict LNM in GC patients with a significantly higher sensitivity of 89.19 than did individual miRNA markers. Thus, these data suggested that the combined detection of miR-27b, miR-128, miR-100 and miR-214 had more considerable clinical value to predict LNM in GC patients. Furthermore, high-risk scores of this 4-miRNA signature tended to have advanced TNM classification for GC patients compared to those with low-risk scores.

Also, univariate Cox regression demonstrated that miR-27b, miR-214 and 4 clinical features, such as LNM, tumor size, clinical stage and TNM were significant survival predictors for GC patients. Consequently, multivariate Cox regression suggested that miR-214 and N stage (TNM classification) were independent prognostic factors for OS. Thus, we developed a 2-miRNA signature (miR-27b and miR-214) and miRNA/N stage signature that were predictive of OS in GC patients. The ROC curve of the 2-miRNA signature or miR-214/N stage had higher AUC than did individual markers. The combination of miRNAs (miR-27b and miR-214) or miR-214/N stage better predicted survival than did individual markers with respect to OS. These results may add supportive evidence that miRNAs have roles for prediction of LNM in GC patients and may improve our understanding of clinical cancer progression and prognosis of GC.

At this time, studies indicate a relationship between 4 miRNAs and LNM or patient prognosis in various human cancers. However, no report is available for miR-27b, miR-128, miR-100 and miR-214 as a signature for predicting LNM in patients with human cancers or the integration of miR-27b and miR-214 or miR-214/N stage as a signature for predicting patient prognosis in human cancers. To our knowledge, this is the first report indicating that the 4-miRNA signature (miR-27b, miR-128, miR-100 and miR-214) possess the prognostic value for LNM in GC patients, contributing to potentially personalized therapy. In addition, our data showed that the 2-miRNA signature or miR-214/N stage could be a useful predictive biomarker for patient’s OS, suggesting a prognostic value for GC patients. However, the present study was limited as only 102 GC patients were involved. The conclusion will need to be validated with large-scale clinical cases in different areas and involving multicenter studies before the 4-miRNA signature and 2-miRNA signature or miR-214/N stage could be clinically applicable for GC patients.

### Ethics approval and consent to participate

The collection of tissue samples was obtained with informed consent and approval to conduct this study was obtained from the Ethics Committee of Shandong University, China.

## Additional Information

**How to cite this article**: Liu, H.-T. *et al*. Prognostic Value of microRNA Signature in Patients with Gastric Cancers. *Sci. Rep.* **7**, 42806; doi: 10.1038/srep42806 (2017).

**Publisher's note:** Springer Nature remains neutral with regard to jurisdictional claims in published maps and institutional affiliations.

## Supplementary Material

Supplementary Information

## Figures and Tables

**Table 1:** Clinical characteristics of patients according to 4-miRNA signatures from GC training and testing sets.

Variable	n	The 4-miRNA signature		*p* value
		Higher risk	Lower risk	
Age (years)				*p* = 0.0333
<60.5	51	15	36	
≥60.5	51	24	27	
Gender				*p* = 0.1143
male	89	36	53	
female	13	3	10	
Tumor size (cm)				*p* = 0.3535
<4.6	42	16	26	
≥4.6	55	23	32	
Missing	5	0	5	
T classification				*p* = 0.0161
T1	10	1	9	
T2	53	17	36	
T3	33	19	14	
T4	3	2	1	
Missing	3	0	3	
Lymph node metastasis				*p* = 0.0003
N0	35	4	31	
N1	44	25	19	
N2	14	6	8	
N3	6	4	2	
Missing	3	0	3	
Distant metastasis (M)				*p* = 0.1633
Negative (M0)	68	29	39	
Positive (M1)	31	10	21	
Missing	3	0	3	
Differentiation				*p* = 0.5697
well	3	2	1	
moderate	31	11	20	
poor	67	26	41	
Missing	1	0	1	

**Table 2:** Univariate and multivariable Cox regression analysis of miRNA expression and OS in training and GC testing sets.

Variable	HR	Univariate analysis		HR	Multivariate analysis	
		CI (95%)	p-value		CI (95%)	p-value
OS						
miR-27b	0.554	0.324–0.946	0.030	0.75	0.336–1.673	0.482
miR-214	0.569	0.333–0.971	0.039	0.363	0.148–0.891	0.027
LNM	4.986	2.187–8.756	0.000	1.378	0.562–3.38	0.483
tumor size	1.821	1.050–3.158	0.033	0.963	0.488–1.9	0.913
clinical stage	6.35	3.330–12.111	0.000	2.317	0.73–7.353	0.154
T stage	1.69	1.152–2.481	0.007	1.873	0.923–3.02	0.082
N stage	3.87	2.128–7.038	0.000	3.304	1.511–7.226	0.003
M stage	4.736	2.659–8.435	0.000	2.648	0.791–8.864	0.114
