# Supplementary material for: Prognostic Value of microRNA Signature in Patients with Gastric Cancers
Source: Sci Rep. 2017 Feb 16;7:42806. doi: 10.1038/srep42806 (PMC5311868; doi:10.1038/srep42806)
Supplement: Supplementary Information [file srep42806-s1.pdf]

Title: Prognostic Value of microRNA Signature in Patients with Gastric Cancers

Hai-Ting Liu[1, 2], Ya-Wen Wang[1, 2], Ai-Yan Xing[1, 2], Duan-Bo Shi[1, 2] , Hui-Zhang[1], Xiang-Yu Guo[1, 2], Jing-Xu[2,3], Peng Gao[1, 2 *]

1 Department of Pathology, Qilu Hospital, Shandong University, Jinan, P.R. China

2 Department of Pathology, School of Medicine, Shandong University, Jinan, P.R. China

3 Department of Pathology, Qingdao Central Hospital, Qingdao, P.R. China.

Supplementary Table 1: The correlation between the six single miRNA expression level and clinicopathologic characteristics of patients in training set of gastric cancers.

		miR-27b expression			miR-101 expression			miR-128 expression			miR-100 expression			miR-145 expression			miR-214 expression		
	n	lower	higher	*p* value	lower	higher	*p* value	lower	higher	*p* value	lower	higher	*p* value	lower	higher	*p* value	lower	higher	*p* value
Age (years)				*p* = 0.0604			*p* = 0.3339			*p* = 0.3339			*p* = 0.3339			*p* = 0.0604			*p* = 0.0604
<61	24	9	15		11	13		11	13		11	13		10	14		9	15	
≥61	27	16	11		14	13		14	13		14	13		15	12		16	11	
Gender				*p* = 0.0457			*p* = 0.0457			*p* = 0.4796			*p* = 0.2066			*p* = 0.0457			*p* = 0.0457
male	45	24	21		24	21		22	23		23	22		24	21		24	21	
female	6	1	5		1	5		3	3		2	4		1	5		1	5	
Tumor size(cm)				*p* = 0.0783			*p* = 0.0395			*p* = 0.0179			*p* = 0.0783			*p* = 0.0395			*p* = 0.0008
<4.5	20	8	12		7	13		6	14		8	12		7	13		5	15	
≥ 4.5	28	17	11		17	11		17	11		17	11		17	11		20	8	
Missing	3	0	3		1	2		2	1		0	3		1	2		0	3	
T classification				*p* = 0.1884			*p* = 0.2294			*p* = 0.3550			*p* = 0.0941			*p* = 0.0365			*p* = 0.0203
T1	6	1	5		2	4		1	5		2	4		2	4		0	6	
T2	26	11	15		13	13		13	13		11	15		9	17		12	14	
T3	15	10	5		10	5		9	6		11	4		12	3		10	5	
T4	2	1	1		0	2		1	1		2	0		1	1		2	0	
Missing	2	2	0		0	2		1	1		0	2		1	1		1	1	
Lymph node metastasis				*p* = 0.3259			*p* = 0.4363			*p* = 0.1614			*p* = 0.1211			*p* = 0.9782			*p* = 0.2578
N0	19	6	13		7	12		6	13		6	13		9	10		6	13	
N1	19	10	9		12	7		11	8		11	8		9	10		11	8	
N2	9	6	3		5	4		5	4		7	2		5	4		6	3	
N3	2	1	1		1	1		2	0		1	1		1	1		1	1	
Missing	2	2	0		0	2		1	1		0	2		1	1		1	1	
Distant metastasis (M)				*P* = 0.1169			*P* = 0.3430			*P* = 0.0812			*P* = 0.0542			*P* = 0.0229			*P* = 0.2129
Negative (M0)	32	17	15		17	15		18	14		19	13		19	13		17	15	
Positive (M1)	17	6	11		8	9		6	11		6	11		5	12		7	10	
Missing	2	2	0		0	2		1	1		0	2		1	1		1	1	
Differentiation				*P* = 0.8892			*P* = 0.8892			*P* = 0.3550			*P* = 0.3270			*P* = 0.2343			*P* = 0.3654

well	2	1	1	1	1	0	2	2	0	2	0	0	2
moderate	18	8	10	8	10	9	9	8	10	7	11	9	9
poor	31	16	15	16	15	16	15	15	16	16	15	16	15

Supplementary Table 2: The correlation between the six single miRNA expression level and clinicopathologic characteristics of patients in testing set of gastric cancers.

		miR-27b expression			miR-101 expression			miR-128 expression			miR-100 expression			miR-145 expression			miR-214 expression		
Variable	n	lower	higher	*p* value	lower	higher	*p* value	lower	higher	*p* value	lower	higher	*p* value	lower	higher	*p* value	lower	higher	*p* value
Age (years)				*p* = 0.2410			*p* = 0.1641			*p* = 0.2410			*p* = 0.2410			*p* = 0.2410			*p* = 0.1032
<59	25	11	14		14	11		11	14		11	14		11	14		10	15	
≥59	26	14	12		11	15		14	12		14	12		14	12		15	11	
Gender				*p* = 0.3627			*p* = 0.3217			*p* = 0.3627			*p* = 0.1220			*p* = 0.1220			*p* = 0.1220
male	44	22	22		21	23		22	22		23	21		23	21		23	21	
female	7	3	4		4	3		3	4		2	5		2	5		2	5	
Tumor size(cm)				*p* = 0.1611			*p* = 0.2543			*p* = 0.4671			*p* = 0.4345			*p* = 0.0585			*p* = 0.0585
<4.5	21	12	9		11	10		10	11		11	10		8	13		10	11	
≥ 4.5	28	12	16		12	16		13	15		14	14		17	11		15	13	
Missing	2	1	1		2	0		2	0					0	2				
T classification				*p* = 0.5648			*p* = 0.5204			*p* = 0.5204			*p* = 0.1153			*p* = 0.1538			*p* = 0.5204
T1	4	1	3		3	1		1	3		0	4		0	4		1	3	
T2	27	14	13		14	13		13	14		16	11		14	13		13	14	
T3	18	9	9		8	10		10	8		9	9		10	8		10	8	
T4	1	1	0		0	1		1	0		0	1		1	0		1	0	
Missing	1	0	1		0	1		0	1		0	1		0	1		0	1	
Lymph node metastasis				*p* = 0.0083			*p* = 0.2839			*p* = 0.0267			*p* = 0.0083			*p* = 0.0716			*p* = 0.0716
N0	16	4	12		8	8		4	12		4	12		5	11		4	12	
N1	25	16	9		10	15		15	10		16	9		16	9		16	9	
N2	5	1	4		4	1		2	3		1	4		1	4		2	3	
N3	4	4	0		3	1		4	0		4	0		3	1		3	1	
Missing	1	0	1		0	1		0	1		0	1		0	1		0	1	
Distant metastasis (M)				*P* = 0.5000			*P* = 0.2644			*P* = 0.2644			*P* = 0.2644			*P* = 0.2644			*P* = 0.5000
Negative (M0)	36	18	18		17	19		17	19		18	18		19	17		18	18	
Positive (M1)	14	7	7		8	6		8	6		7	7		6	8		7	7	
Missing	1	0	1		0	1		0	1		0	1		0	1		0	1	
Differentiation				*P* = 0.4460			*P* = 0.4460			*P* = 0.4460			*P* = 0.5521			*P* = 0.5836			*P* = 0.1857
well	1	1	0		1	0		1	0		1	0		1	0		1	0	
moderate	13	5	8		5	8		5	8		7	6		6	7		4	9	
poor	36	18	18		18	18		18	18		17	19		18	18		20	16	
Missing	1	1	0		1	0		1	0		0	1		0	1		0	1	

Supplementary Table 3: The correlation between the six single miRNA expression level and clinicopathologic characteristics of patients in combination of training set and testing set of gastric cancers

		miR-27b expression			miR-101 expression			miR-128 expression			miR-100 expression			miR-145 expression			miR-214 expression	
Variable	n	lower	higher	*p* value	lower	higher	*p* value	lower	higher	*p* value	lower	higher	*p* value	lower	higher	*p* value	lower	higher
Age (years)				p = 0.0828			p = 0.4215			p = 0.1611			p = 0.1611			p = 0.0828		
<60.5	51	22	29		25	26		23	28		23	28		22	29		31	20
⩾60.5	51	29	22		26	25		28	23		28	23		29	22		20	31
Gender				p = 0.0688			p = 0.3833			p = 0.3833			p = 0.1865			p = 0.0188		
male	89	47	42		44	45		45	44		46	43		48	41		48	41
female	13	4	9		7	6		6	7		5	8		3	10		3	10
Tumor size(cm)				p = 0.1387			p = 0.1676			p = 0.0372			p = 0.2494			p = 0.0032		
<4.5	42	19	23		18	24		16	26		20	22		15	27		15	27
≥ 4.5	55	31	24		29	26		31	24		30	25		35	20		36	19
Missing	5	1	4		4	1		4	1		1	4		1	4		0	5
T classification				p = 0.2391			p = 0.1857			p = 0.1430			p = 0.1037			p = 0.0108		
T1	10	2	8		4	6		2	8		3	7		2	8		1	9
T2	53	27	26		27	26		26	27		25	28		23	30		25	28
T3	33	18	15		20	13		20	13		20	13		22	11		20	13
T4	3	2	1		0	3		2	1		3	0		3	0		3	0
Missing	3	2	1		0	3		1	2		0	3		1	2		1	2
Lymph node metastasis				p = 0.0055			p = 0.1568			p = 0.0065			p = 0.0135			p = 0.2842		
N0	35	10	25		14	21		11	24		11	24		14	21		10	25
N1	44	28	16		23	21		26	18		28	16		26	18		28	16
N2	14	6	8		9	5		7	7		7	7		6	8		8	6
N3	6	5	1		5	1		6	0		5	1		4	2		4	2
Missing	3	2	1		0	3		1	2		0	3		1	2		1	2
Distant metastasis (M)				P = 0.4408			P = 0.4774			P = 0.1764			P = 0.1427			P = 0.0368		
Negative (M0)	68	34	34		35	32		36	31		37	30		38	29		36	31
Positive (M1)	31	15	16		16	16		14	18		14	18		13	20		14	18
Missing	3	2	1		0	3		1	2		0	3		1	2		1	2
Differentiation				P = 0.7302			P = 0.8308			P = 0.6879			P = 0.1925			P = 0.1401		
well	3	2	1		2	1		1	2		3	0		3	0		1	2
moderate	31	14	17		15	16		14	17		14	17		13	18		12	19
poor	67	34	33		33	34		35	32		33	34		35	32		38	29
Missing	1	1	0		1	0		1	0		1	0		0	1		0	1

Supplementary Table 4: Performance of miR-27b, miR-128, miR-100, miR-214 and the 4 miRNA-signature for detection of LNM in gastric cancer patients.

variables	AUC(95%CI)	p value	Sensitivity (%)	Specificity (%)
miR-27b	0.734(0.631-0.838)	0.000	80%	62.16%
miR-128	0.719(0.616-0.822)	0.000	67.69%	70.27%
miR-100	0.741(0.647-0.835)	0.000	60%	83.78%
miR-214	0.731(0.632-0.829)	0.000	44.62%	94.59%
logit(Y)	0.760(0.665-0.854)	0.000	89.19%	53.85%

Supplementary Table 5: Performance of miR-27b, miR-214 and the 2 miRNA-signature for detection of patients' overall survival in gastric cancers.

variables	AUC(95%CI)	p value	Sensitivity (%)	Specificity (%)
miR-27b	0.618(0.508-0.729)	0.040	87.29%	36.17%
miR-214	0.614(0.505-0.723)	0.048	83.64%	38.30%
the 2-miRNA signature	0.630(0.521-0.739)	0.024	89.09%	34.04%

Supplementary Table 6: Clinical characteristics of patients according to the 4-miRNA signature in training set of gastric cancers.

		the 4-miRNA signature		
Variable	n	higher risk	lower risk	*p* value
Lymph node metastasis				*p* = 0.0196
Yes	31	20	11	
No	20	7	13	
Age (years)				*p* = 0.1688
<61	24	11	13	
⩾61	27	16	11	
Gender				*p* = 0.0290
male	45	26	19	
female	6	1	5	
Tumor size(cm)				*p* = 0.0006
< 4.5	21	6	15	
⩾ 4.5	28	21	7	
Missing	2	0	2	
T classification				*p* = 0.2289
T1	6	1	5	
T2	26	14	12	
T3	15	10	5	
T4	2	1	1	

Missing	2	1	1	
Lymph node metastasis				*p* = 0.2214
N0	19	7	12	
N1	19	11	8	
N2	9	7	2	
N3	2	1	1	
Missing	2	1	1	
Distant metastasis (M)				*P* = 0.4951
Negative (M0)	32	17	15	
Positive (M1)	17	9	8	
Missing	2	0	2	
Differentiation				*P* = 0.6521
well	2	1	1	
moderate	18	8	10	
poor	31	18	13	

Supplementary Table 7: Clinical characteristics of patients according to the 4-miRNA signature in testing set of gastric cancers.

		the 4-miRNA signature		
Variable	n	higher risk	lower risk	*p* value
Lymph node metastasis				*p* = 0.0147
Yes	34	21	13	
No	17	5	12	
Age (years)				*p* = 0.3382
<59	25	12	13	
≥59	26	14	12	
Gender				*p* = 0.3217
male	44	23	21	
female	7	3	4	
Tumor size(cm)				*p* = 0.4443
< 5	24	12	12	
≥ 5	25	13	12	
Missing	2	1	1	
T classification				*p* = 0.5208
T1	4	1	3	
T2	27	15	12	
T3	18	9	9	
T4	1	1	0	
Missing	1	0	1	
Lymph node metastasis				*p* = 0.0190
N0	16	5	11	
N1	25	16	9	
N2	5	1	4	

N3	4	4	0	
Missing	1	0	1	
Distant metastasis (M)				*P* = 0.4508
Negative (M0)	35	18	17	
Positive (M1)	15	8	7	
Missing	1	0	1	
Differentiation				*P* = 0.5836
well	1	1	0	
moderate	13	6	7	
poor	36	18	18	
Missing	1	0	1	

Supplementary Table 8: Performance of miR-214, N stage and the miR-214/ N stage signature for detection of patients' overall survival in gastric cancers.

variables	AUC(95%CI)	p value	Sensitivity (%)	Specificity (%)
miR-214	0.614(0.505-0.723)	0.048	72.73%	40.43%
N stage	0.511(0.439-0.633)	0.376	70.2%	60.0%
the miR-214/N stage signature	0.6627 (0.5560-0.7693)	0.00477	72.73%	57.45%

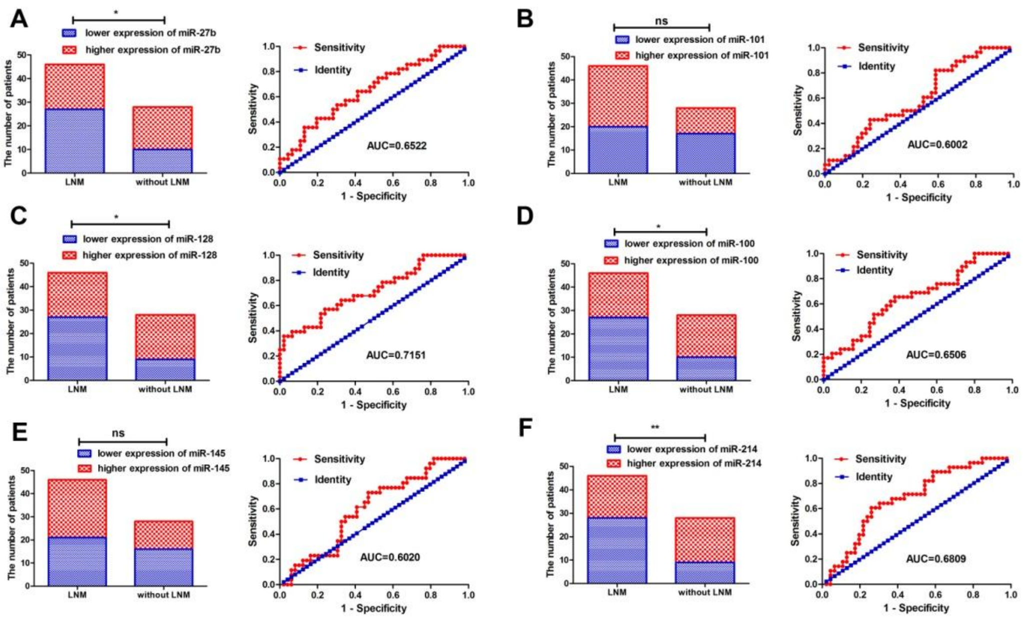

Supplementary Figure Legend

Figure 1: The expression level of the six cancer-related miRNAs and comparison of accuracy for the prediction of patients with LNM in 74 gastric cancer samples. The expressions of miR-27b (A), miR-128 (C), miR-100 (D) and miR-214 (F) in the patients with LNM were significantly lower than that in the patients without LNM (all

P < 0.05).No correlation was found between the expression levels of miR-101 (B) and miR-145 (E) and LNM (all P > 0.05). Area under the curve (AUC) for the miRNAs. A: miR-27b (P = 0.02897); B: miR-101 (P = 0.1506); C: miR-128 (P = 0.0020); D: miR-100 (P = 0.02962); E: miR-145 (P = 0.1479); F: miR-214 (P = 0.0094). RNU44 was used as the endogenous control to normalize the relative expression of miRNA.
